# Oncocytic Tumors: An Uncommon Benign Adrenal Gland Lesions With Features of Malignancy

**DOI:** 10.7759/cureus.33638

**Published:** 2023-01-11

**Authors:** Zineb Elazime, Lamiaa Elazizi, Hayat Aynaou, Houda Salhi, Hanan Elouahabi

**Affiliations:** 1 Department of Endocrinology, Diabetology, Metabolic Diseases and Nutrition, Hassan II University Hospital Center, Fez, MAR

**Keywords:** rare tumors, adrenal incidentaloma, malignancy, adrenal oncocytoma, oncocytoma

## Abstract

Oncocytic cell neoplasms are usually found in the thyroid or salivary glands and the kidneys. Adrenal oncocytoma (AO) is an extremely rare localization. It is often non-functional and the suspicion of malignancy is considered when the size of an adrenal incidentaloma is greater than 4 cm. These adrenal oncocytomas, however, are large, round, and encapsulated with a benign presentation and evolution.

We report on a 40-year-old male patient. Upon suspicion of a SARS-CoV-2 infection, the general physician instructed a chest CT scan, which, fortuitously, revealed a suspicious left adrenal lesion measuring 62x45mm. A biochemical investigation was negative for either pheochromocytoma or Cushing's syndrome, allowing the recommended surgery to be performed. The anatomopathological analysis showed an uncommon benign adrenal lesion, an adrenal oncocytoma.

## Introduction

Adrenal oncocytoma is an extremely rare tumor. It was originally reported by Kakimoto et al. [1] in 1986. Since then, less than 200 cases of adrenal oncocytoma have been reported [2,3].

Oncocytoma is an epithelial neoplasm consisting of cells with an eosinophilic cytoplasm and rich in mitochondria. They can occur in different organs, including the kidneys, the pituitary, the thyroid, the salivary, and the parathyroid glands [4]. Rare cases have been documented in the respiratory and gastrointestinal tracts [5].

The diagnosis of adrenal oncocytomas is commonly incidental since they are often nonfunctional; however, biochemical testing is suggested to rule out the functional form. Imaging is not a valuable diagnostic method for oncocytomas because they may have characteristics that are seen commonly in other tumors, like adrenal carcinoma [6].

As oncocytomas are likely benign tumors, the diagnosis has important implications for monitoring. However, there is limited data on monitoring strategies [7].

Herein, we present the case of a 40-year-old male patient whose chest CT scan showed a suspicious adrenal lesion. The patient underwent surgery after pheochromocytoma and Cushing’s syndromes were ruled out. The lesion finally turned out to be an oncocytoma, a rare benign lesion.

## Case presentation

**Figure 1:**
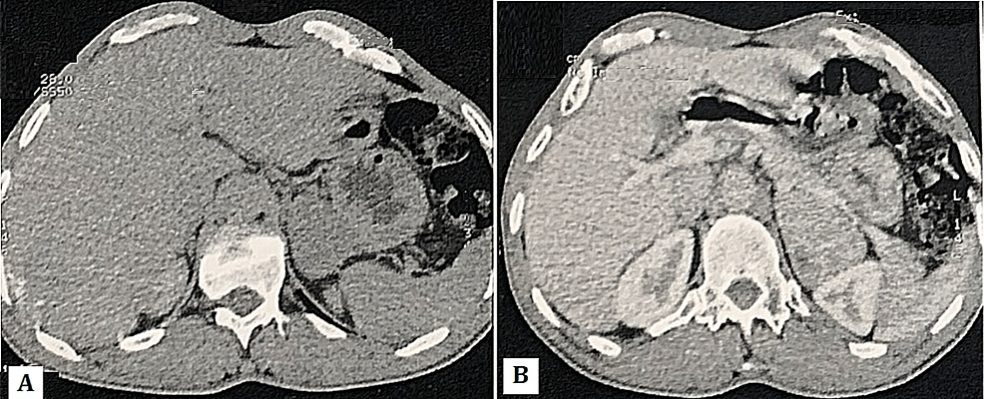
(A) Adrenal CT, axial section: left adrenal lesion, solid-necrotic with a spontaneous density of 40UH. (B) Axial image on portal phase (CT) scan showing a left adrenal mass, heterogeneously enhanced

We report the case of a 40-year-old male patient with no significant medical history. In 2021, the patient complained to his family physician of a flu-like syndrome; a chest CT scan was performed to rule out a SARS-CoV-2 infection, which revealed a suspicious adrenal lesion. The patient was referred to our department for adrenal incidentaloma, where we performed a hormonal analysis to check for the potential of a functional mass and a repeat chest CT scan. The latter showed a left adrenal lesion measuring 62 x 45 mm, solid-necrotic with a spontaneous density of 40UH, 84% absolute washout, and 37% relative washout (Figure 1A-1B).

**Figure 2:**
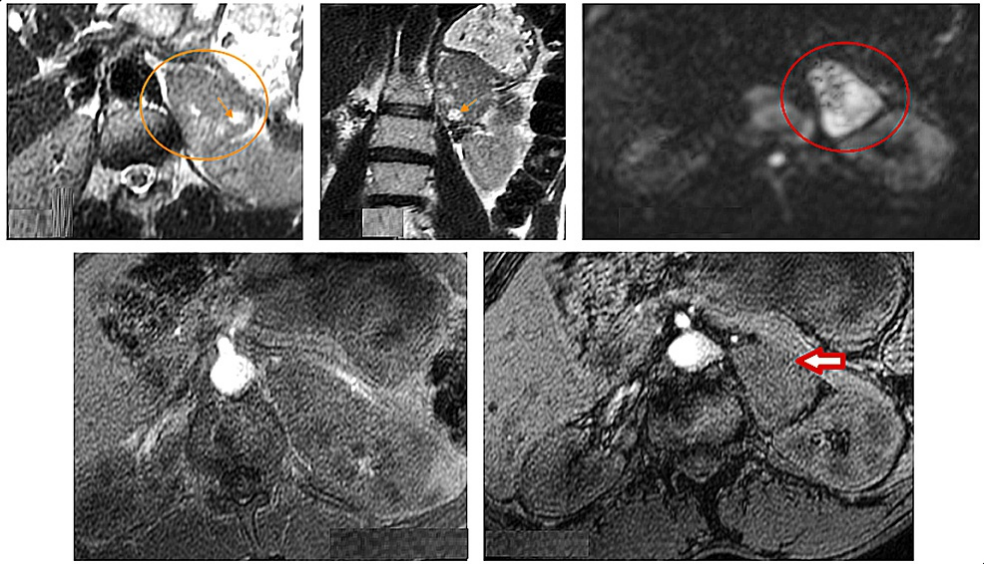
Voluminous mass in the external part of the left adrenal gland, well limited, with regular contours (orange circle), encapsulated, described in intermediate T2 signal with areas in marked T2 hypersignal (orange arrow), restrictive in diffusion (red circle), not showing any signal decrease in the OP sequences (red arrow)

**Figure 3:**
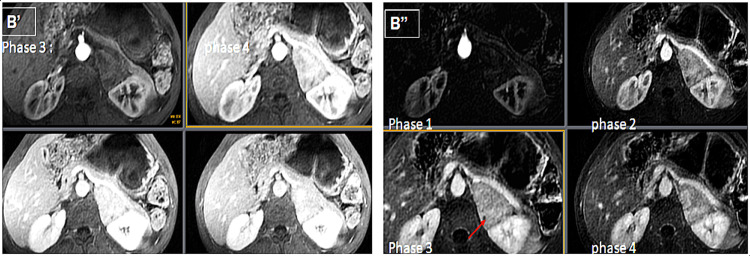
Heterogeneous and progressive enhancement following a type III enhancement curve, (non-subtracted series B' / subtracted series B''), delimiting areas of necrosis (red arrow).

Considering that the patient had neither hypertension nor hypokalemia, we wanted to rule out pheochromocytoma and Cushing's syndrome. The results of this study are as follows: a negative urinary fractionated metanephrines test, 1 mg dexamethasone overnight of 0.6ug/dl µg/dl. In the presence of atypical features of the adrenal lesion on the chest CT scan, an MRI was performed and detected a large mass in the external portion of the left adrenal gland. The mass was well limited with regular contours, encapsulated with intermediate T2 signal with areas of high T2 signal, restrictive in diffusion, without signal drop in out-of-phase (OOP) sequences, and with progressive heterogeneous enhancement following a type III enhancement curve, outlining areas of necrosis (Figures 2-3). It measured 59 x 43 mm, responsible for the reflow of the external splenic vein, without any signs of kidney or pancreas invasion.

The patient underwent a left adrenalectomy via laparotomy and had an uneventful recovery.

The histological analysis revealed large polygonal cells with abundant granular and eosinophilic cytoplasm and a vesicular nucleus without cytonuclear atypia. The immuno-histological analysis showed an intense and diffuse marking of anti-synaptophysin and anti-MelanA antibodies, anti-chromogranin antibodies, and cytokeratin, which were all negative. The proliferation index evaluated by Ki67 is less than 5% (2%). Besides, the PS100 antibody showed the presence of scattered star cells. Hence, the diagnosis of adrenocortical oncocytoma was made.

A month after the surgery, the patient was completely asymptomatic, with no evidence of surgical wound infection or incisional hernia. He is now under follow-up.

## Discussion

The oncocytic cells have been described initially in 1931 by Hamperl as cells with a granular and eosinophilic cytoplasm [3]; these characteristics were the consequence of several mitochondria [8]. Although the mechanisms of oncocytosis are not fully known, two theories exist to explain it: the first one is that the proliferation of mitochondria is the consequence of a mutation and the second one is the result of an epigenetic event resulting from the cellular hypoxia [9].

Adrenal oncocytoma is extremely rare; nearly 200 cases have been published since Kakimoto et al. reported the first case [1]. They affect a wide age group (15-77 years), more prevalent in women and in the left adrenal gland [10]. They usually manifest as an incidentaloma.

According to current recommendations, an adrenal incidentaloma greater than 4 cm and over 10 HU is presumptively malignant. Despite this fact, an adrenal oncocytoma is usually benign. Approximately, 25% of AOs reported in the literature are malignant, but in some recent series, a percentage greater than 60-70% have been found [11]. Also, AO has been reported to be associated with hormonal hypersecretion in 31.5% of cases, principally as a Cushing's syndrome, virilizing syndrome, or pheochromocytoma-like syndrome [12].

Imaging techniques (e.g., CT, MRI) have limited sensitivity for adrenal oncocytoma because it is hardly distinguishable from a malignant tumor. Basically, AOs are in general large (8.5 cm on average), low in lipids, with increased attenuation on the CT from 20 to 40 HU, and have malignant features such as heterogeneous contrast enhancement or fibrous encapsulation.

Macroscopically, the AO is a well-circumscribed enclosed lesion, in which areas of bleeding and/or necrosis can be detected. The defining histological characteristic of these tumors is a proliferating oncocytic cell, with an increased size and an eosinophilic granular cytoplasm, due to the accumulation of mitochondria. They are usually positive for vimentin, calretinin, alpha-calretinin, alpha-inhibin, and melanin-A and negative for S100 and chromogranin [13].

In terms of prognosis, while the Weiss criteria [14] are used to establish the malignant behavior of adrenocortical carcinomas, these standards are not appropriate for AO. Thus, for better stratification, the Lin-Weiss-Bisceglia system has been proposed for AO [15], which distinguishes major criteria (>5 mitoses/50 hpf, presence of atypical mitoses or venous invasion) and minor criteria (height >10 cm or weight >200 g, presence of tumor necrosis, capsular or sinusoidal invasion). The presence of any major criteria would classify the tumor as malignant, while minor criteria, such as a lesion of uncertain potential, and the absence of all criteria would suggest a benign lesion.

Concerning the treatment of these tumors, their association with hormonal hypersecretion and their suspicion of malignancy make surgery the first option. Adrenalectomy is usually performed by laparotomy for large tumors or by laparoscopy for smaller ones if imaging shows a well-encapsulated tumor with no invasion.

There are few recommendations for follow-up. No recurrent benign or indeterminate oncocytomas have been reported. Patients with malignant oncocytomas, however, have a five-year survival rate of 50-60% after surgery. Out of the nine cases of malignant oncocytoma, five were reported disease-free in six months, whereas four experienced a recurrence [3].

It is recommended that regular monitoring should be maintained for at least five years [3,7].

## Conclusions

Adrenal oncocytoma is a rare tumor. Surgical removal remains the primary method of treatment and it can be considered in most cases as a benign tumor. This case report is a valuable contribution to understanding adrenal oncocytoma due to the lack of information based on cases reports. However, large clinical studies are much needed to confirm the results as well as in order to evaluate the prognosis and establish follow-up strategies.
